# Development of an Antiviral Ion-Activated In Situ Gel Containing 18β-Glycyrrhetinic Acid: A Promising Alternative against Respiratory Syncytial Virus

**DOI:** 10.3390/pharmaceutics15082055

**Published:** 2023-07-31

**Authors:** Burcu Özkan, Ebru Altuntaş, Ümmühan Ünlü, Hasan Hüseyin Doğan, Yıldız Özsoy, Rabia Çakır Koç

**Affiliations:** 1 Graduate School of Natural and Applied Science, Yildiz Technical University, Istanbul 34220, Turkey; burcu_ozkan93@hotmail.com; 2 Faculty of Pharmacy, Department of Pharmaceutical Technology, Istanbul University, Istanbul 34116, Turkey; yozsoy@istanbul.edu.tr; 3 Elderly Care Program, Ataturk Health Services Vocational School, Afyonkarahisar Health Sciences University, Afyonkarahisar 03030, Turkey; ummuhan.unlu@afsu.edu.tr; 4 Department of Biology, Science Faculty, Alaeddin Keykubat Campus, Selcuk University, Konya 42130, Turkey; hhdogan@selcuk.edu.tr; 5 Faculty of Chemical and Metallurgical Engineering, Department of Bioengineering, Yildiz Technical University, Istanbul 34220, Turkey; rabiakoc@yildiz.edu.tr

**Keywords:** in situ gelling formulations, intranasal delivery, gellan gum, mucoadhesion, 18β-glycyrrhetinic acid, respiratory syncytial virus, common cold, antiviral activity

## Abstract

The human respiratory syncytial virus (hRSV) is a major cause of serious lower respiratory infections and poses a considerable risk to public health globally. Only a few treatments are currently used to treat RSV infections, and there is no RSV vaccination. Therefore, the need for clinically applicable, affordable, and safe RSV prevention and treatment solutions is urgent. In this study, an ion-activated in situ gelling formulation containing the broad-spectrum antiviral 18β-glycyrrhetinic acid (GA) was developed for its antiviral effect on RSV. In this context, pH, mechanical characteristics, ex vivo mucoadhesive strength, in vitro drug release pattern, sprayability, drug content, and stability were all examined. Rheological characteristics were also tested using in vitro gelation capacity and rheological synergism tests. Finally, the cytotoxic and antiviral activities of the optimized in situ gelling formulation on RSV cultured in the human laryngeal epidermoid carcinoma (HEp-2) cell line were evaluated. In conclusion, the optimized formulation prepared with a combination of 0.5% *w*/*w* gellan gum and 0.5% *w*/*w* sodium carboxymethylcellulose demonstrated good gelation capacity and sprayability (weight deviation between the first day of the experiment (T0) and the last day of the experiment (T14) was 0.34%), desired rheological synergism (mucoadhesive force (Fb): 9.53 Pa), mechanical characteristics (adhesiveness: 0.300 ± 0.05 mJ), ex vivo bioadhesion force (19.67 ± 1.90 g), drug content uniformity (RSD%: 0.494), and sustained drug release over a period of 6 h (24.56% ± 0.49). The optimized formulation demonstrated strong anti-hRSV activity (simultaneous half maximal effective concentration (EC_50_) = 0.05 µg/mL; selectivity index (SI) = 306; pre-infection EC_50_ = 0.154 µg/mL; SI = 100), which was significantly higher than that of ribavirin (EC_50_ = 4.189 µg/mL; SI = 28) used as a positive control against hRSV, according to the results of the antiviral activity test. In conclusion, this study showed that nasal in situ gelling spray can prevent viral infection and replication by directly inhibiting viral entry or modulating viral replication.

## 1. Introduction

Respiratory viruses lead to the majority of respiratory tract infections, which are the primary cause of morbidity and mortality in humans [1]. As the most frequent source of symptoms-based illness that results in a substantial financial burden from an increase in sick days, respiratory virus infections are a concern for global public health [2,3]. The most common viruses involved are adenoviruses; parainfluenza types 1, 2, and 3; respiratory syncytial virus (RSV); and influenza A and B [4].

RSV, a non-segmented negative-strand, enveloped virus, is a member of the *Paramyxoviridae* family of RNA viruses. In terms of the number of genes and proteins, RSV is the most sophisticated member of the family [5]. Infection with RSV poses a considerable risk to the elderly and is one of the most common causes of serious respiratory disease in newborns and young children globally [6]. RSV is a virus that can potentially lead to the common cold [7]. RSV was first identified in chimpanzees who had cold-like symptoms in 1955 [8]. In the years that followed, the virus was also isolated from newborns suffering from serious lower respiratory tract diseases [9]. Since then, research has established that RSV is a common disease affecting almost all children, with half of them acquiring two infections during this time [10]. RSV has also been linked to chronic respiratory diseases including asthma, idiopathic pulmonary fibrosis, obstructive pulmonary disease, and chronic bronchitis. Furthermore, it can have a major negative impact on the elderly, particularly those with weakened immune systems, chronic bronchitis, and other medical conditions, including chronic obstructive pulmonary disease [11].

RSV infections are typically transmitted through intimate contact; however, they can also be conveyed through aerosolized droplets into the environment [12]. Few methods for preventing or treating RSV infection have been established, despite years of continuous work. Since RSV was discovered to be a human infection more than 60 years ago, disappointingly, no approved vaccine has yet been discovered [12]. The inadequate immunological response of humans to RSV is one explanation for this. For instance, two months after a prior infection, adult participants may be infected with RSV again [6]. Only two RSV antiviral medications have been given FDA approval to treat or prevent serious RSV-related respiratory tract infections: aerosolized ribavirin for treatment and palivizumab (Synagis^®^) for prophylaxis [13]. A broad-spectrum antiviral drug with efficacy against RSV and other RNA viruses, including hepatitis C and the Zika virus, is the guanosine analog ribavirin [14,15,16]. A number of studies have shown that this medication has a positive impact on preventing RSV replication. Infected cotton rats with RSV lung titers were treated with ribavirin, which demonstrated antiviral efficacy against RSV [17]. Similar to adults, children treated with aerosolized ribavirin in the early infection stage have shown considerable clinical improvements [18]. However, ribavirin’s nonspecific anti-RSV efficacy, high potential for toxicity, and relatively expensive cost limit its use in practice [19]. Additionally, ribavirin has not demonstrated a significant effect on clinically important outcomes such as mortality, hospital stay length, the need for mechanical breathing, or admission to an intensive care unit [20,21,22]. Palivizumab, a humanized monoclonal antibody (mAb), is the only immunoprophylactic agent for serious lower respiratory tract infection caused by RSV that has received FDA approval in certain high-risk pediatric populations, such as newborns born at or under 35 weeks gestational age (wGA), children with severe hemodynamic congenital heart disease, and children with premature chronic lung disease [21]. Palivizumab is solely advised for use as a preventative measure; it is not recommended for the management of RSV infection. Data show that once an RSV infection has occurred, it has had no impact on the results [23]. Palivizumab has been shown to be both effective and safe for preventing RSV infection in pediatric populations at high risk in randomized, post-licensure efficacy studies and placebo-controlled trials [24,25]. Nevertheless, the high cost, the short half-life requiring monthly injections, and a tight RSV immunoprophylaxis guideline from the American Academy of Pediatrics are a few obstacles preventing palivizumab use in compliance with its license [26]. The demand for clinically viable, safe, and cost-effective RSV prevention and treatment alternatives is therefore paramount.

Herbal antiviral agents may be a potential alternative in humans for respiratory viruses for therapeutic or prophylactic purposes. In fact, “18β-glycyrrhetinic acid (GA)”, a broad-spectrum potent antiviral herbal agent, is a pentacyclic triterpenoid that is the key metabolite of glycyrrhizic acid, the main water-soluble component of licorice root. GA and its derivatives are components of natural origin with a wide spectrum of bioactivity, including antitumor [27,28,29], antiviral [30], antimicrobial [31], anti-ulcer [32], antidiabetic [33], hepatoprotective [34], cardioprotective, and neuroprotective effects [35]. Hardy et al. demonstrated that GA therapy prevented rotavirus replication, which most likely took place after virus entry. When GA was applied to infected cultures after viral adsorption, it was discovered that the yields of rotavirus were reduced by 99%. The viral proteins VP2, VP6, and NSP2 were significantly downregulated [36]. In addition, the strong hRSV activity of GA has been demonstrated. It largely prevented viral attachment, stimulated interferon (IFN) secretion, and inhibited hRSV internalization. In addition to blocking viral attachment, GA also inhibits viral replication and boosts host cell activity [37].

The viral loads in symptomatic and asymptomatic patients are comparable, and the nasal cavity and nasopharynx have some of the highest viral loads in the body. Given that nasal secretions contain a virus that might transmit and that contagiousness seems to be at its peak before or immediately after symptom onset, these “silent spreaders” may accidentally contribute to the exponential expansion of disease. In order to accomplish treatment goals, intranasal delivery of antiviral medications or agents may present an additional choice for limiting the spread of disease, treating the nasal disease, and supplying perioperative antisepsis [38].

One of the most significant barriers to successful active substance delivery through the nose is mucociliary clearance, as it reduces the drug’s residence time in the zone of action. For nasal applications, the formulation’s contact with the nasal mucosa can be prolonged to maximize effectiveness. For this purpose, mucoadhesive carrier systems are being developed. Increasing the viscosity using mucoadhesive polymers may be beneficial to avoid the formulation draining and to extend the contact time between the nasal mucosa and the drug [39]. The adherence of a polymer to a mucus layer is referred to as mucoadhesion. The mucus layer is a sticky and viscous layer composed primarily of mucin and water [40,41]. The ability of a polymer to bind to the mucus layer depends on several factors, including swelling, molecular weight, and the flexibility of polymer chains, as well as chemical bond formation [42,43]. Compared with conventional liquid nasal formulations, mucoadhesive gels extend the contact time between the nasal mucosa and the active substance [44,45]. However, nasal administration of typical mucoadhesive gels may be technically difficult and problematic for delivering a proper dose of medications due to the high consistency of the formulation. As a result, in situ gelling formulations (also known as environmentally sensitive gels), a novel dosage form employed in nasal medication applications, have recently grown to be quite appealing [46]. Compared to nasal formulations in liquid form, nasal in situ gelling formulations are low-viscosity fluids prior to administration and form a gel by changing the polymer structure after contact with the nasal mucosa. Therefore, in situ gelling formulations not only extend the contact duration between the nasal mucosa and the drug, but also ensure that drug release occurs slowly and continuously. The transition from solution form to gel form (sol–gel phase transition) can occur with a change in pH (e.g., cellulose acetate phthalate), a change in temperature known as thermogelling (e.g., poloxamer 407), or the existence of cations (e.g., gellan gum) [39]. In situ gelling formulations not only have the benefits of a solution, such as ease of application, simplicity of preparation, no foreign body feeling, and complete dosing, but also an increased residence time in the nasal mucosa similar to a gel. These advantages improve treatment efficacy and patient compliance [47].

A deacetylated, anionic, exocellular bacterial polymer called gellan gum was first identified in 1978. The repeating tetrasaccharide units of 1-L-rhamnose, 1-D-glucuronic acid, and 2-D-glucose are released from *Sphigomonas paucimobilis*, formerly referred to as *Pseudomonas elodea*. The development of double-helical junction zones is the first step in the mechanism of gelation. Next, the double-helical segments are aggregated to create a 3D network by complexing with cations and forming hydrogen bonds with water [48]. The type of cations in gellan gum solutions affects their ability to gel, and divalent cations work significantly better than monovalent cations to facilitate gelation. The use of deacetylated gellan gum (DGG) as a stabilizing, gelling, and suspending ingredient in food products is permitted both in the EU and the USA, and is sold under the trade names Gelrite^®^ or Kelcogel^®^. Therefore, gellan gum can be used safely in pharmaceuticals. Furthermore, gellan gum can be used in biomedical technology, including drug delivery systems and as a medium for protein immobilization, and is one of the most intriguing in situ gelling polymers for the human body [39,48,49].

In the current study, a nasal mucoadhesive spray formulation of an ion-activated in situ gelling formulation containing GA with broad-spectrum antiviral activity and an antiviral effect against RSV was developed. Rheological characteristics with in vitro gelation capacity and rheological synergism, mechanical characteristics, sprayability, drug content, pH, ex vivo mucoadhesive strength, in vitro drug release pattern, and stability analyses were examined. Finally, the cytotoxic and antiviral effects of the optimized formulation on RSV cultured in the HEp-2 cell line were tested.

## 2. Materials and Methods

### 2.1. Materials

The 18β-glycyrrhetinic (GA) 97%, dexpanthenol, benzalkonium chloride, and porcine gastric mucin (type II) were purchased from Sigma-Aldrich (St. Louis, MO, USA); Kelcogel^®^ CG-LA (deacetylated gellan gum, DGG) was kindly provided by Azelis (Istanbul, Türkiye); VANZAN^®^ NF (xanthan gum) was kindly provided by Vanderbilt Minerals, LLC (Norwalk, CT, USA); Blanose™ (sodium carboxymethylcellulose, Na-CMC) and Benecel™ E10M (hydroxypropyl methylcellulose, HPMC) were kindly gifted by Ashland (Istanbul, Türkiye), Carbopol^®^ 974P NF was provided as a gift from Lubrizol (Wickliffe, OH, USA); and methanol ≥ 99.9% for liquid chromatography and ortho-phosphoric acid 85% were supplied by Isolab Chemicals (Istanbul, Turkey). Fetal bovine serum (FBS), Dulbecco’s phosphate buffered saline (DPBS), antibiotic-antimycotic solution (100×), ribavirin and minimum essential medium (MEM) were purchased from Sigma-Aldrich (St. Louis, MO, USA); 0.25% Trypsin-EDTA (1×) solution was provided from (Diagnovum, Ebsdorfergrund, Germany); Trypan blue dye was purchased from NutriCulture (Skelmersdale, UK); and the XTT [2,3-Bis- (2-Methoxy-4-Nitro-5-Sulfophenyl)-2H-Tetrazolium-5-Carboxanilide] kit was obtained from Biological Industries Ltd. (Kibbutz Beit Haemek, Israel). All other chemicals and reagents used in the study were of analytical grade.

### 2.2. Methods

#### 2.2.1. Preparation of GA-Loaded In Situ Gelling Formulations

##### Preparation of In Situ Gelling Systems Using Only Gellan Gum

The method according to Morsi et al. was used to prepare GA-loaded in situ gelling formulations (Table 1) [50]. In order to prepare a clear DGG solution, various concentrations of DGG (0.2–1%, *w*/*v*) were added to distilled water and stirred moderately for 20 min at 500 rpm on a 90 °C hot magnetic stirrer for complete dissolution. Thereafter, the cooled DGG solution was blended thoroughly with GA powders to obtain the final formulations. For comfort during administration and to prevent pain and irritation at the administration site, formulations should be isotonic for nasal administration. Glycerin, one of the 5 excipients listed in the United States Pharmacopoeia—National Formulary (USP 44—NF 39) as a tonicity modifier, was used in all formulations at a concentration of 1% (*w*/*w*), according to the literature [51,52]. Moreover, other excipients, including the mucous moisturizer dexpanthenol and the preservative benzalkonium chloride, were added while being continuously stirred. Finally, the mixture was stirred continuously for 24 h.

##### Preparation of In Situ Gelling Systems Using Combined Polymers

In situ gelling systems were prepared by combining DGG with one of four mucoadhesive polymers (xanthan gum, Na-CMC, HPMC, or Carbopol^®^ 974P NF). Deionized water was used to dissolve the polymers in the DGG/xanthan, DGG/HPMC, and DGG/Na-CMC systems by heating them at 90 °C for 20 min and then allowing them to cool to room temperature (RT). In order to prepare the systems containing Carbopol^®^ 974P NF, the polymer was sprinkled in cool deionized water, allowed to hydrate, and then mixed with a DGG solution. The polymer solution was then continuously stirred with GA and additional formulation excipients to achieve a final polymer concentration of 0.5% *w*/*w* (xanthan gum, HPMC, Na-CMC, or Carbopol^®^ 974P NF) together with DGG (0.4, 0.5, or 0.6% *w*/*w*). Finally, the mixture was stirred continuously for 24 h. The chemical compositions of various in situ gelling systems containing DGG (0.4, 0.5, or 0.6% *w*/*w*) and a fixed amount (0.5% *w*/*w*) of mucoadhesive polymers are shown in Table 2.

Lastly, in situ gelling formulations containing different concentrations of Na-CMC as a selected mucoadhesive polymer (0.1, 0.3, 0.5, and 0.7% *w*/*v*) and DGG (0.5% *w*/*v*) were prepared (Table 3), and an in situ gelling formulation–mucin interaction study was conducted on these formulations.

**Figure 1:**
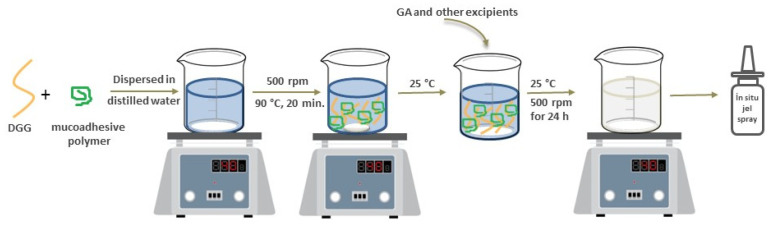
Diagrammatic representation of the fabrication process of GA-loaded in situ gelling formulations using combined polymers except for Carbopol^®^ 974P NF.

GA-loaded in situ gelling formulations were prepared using combined polymers except for Carbopol^®^ 974P NF, as shown in Figure 1 below.

#### 2.2.2. Characterization of GA-Loaded In Situ Gelling Formulations

##### Gelation Capacity of In Situ Gelling Formulations with/without Mucoadhesive Polymers

Formulations (1.0 mL) were mixed with simulated nasal fluid (SNF) (0.5 mL), which contained 7.45 mg/mL NaCl, 0.32 mg/mL CaCl_2_·2H_2_O, and 1.29 mg/mL KCl in transparent glass vials, to measure the gelation capacity of the formulations. These transparent glass vials were then put in a water bath set at 34 °C. After inverting the vial for 20 s, in situ gelation was observed by visual inspection [53,54]. An in situ gelation of the formulation was also demonstrated by rheological analysis using a cone-plate viscometer (Brookfield HA DV3T, Brookfield, UK). Each sample was examined in the viscometer, which had a spindle 52 suitable for gelled systems, at shear rates ranging from 20 to 100 rpm. All measurements were carried out in triplicate [50].

##### Rheological Evaluation

The same cone and plate viscometer was used to determine the viscosity values of the prepared in situ gelling formulations. The viscometer was equipped with a cone spindle 40 for the liquid formulations prior to gelation with the SNF solution. For the case of gelled systems, the viscometer was equipped with spindle 52, and all samples were tested at shear rates of between 20 and 100 rpm (keeping a period of 10 s at each rpm). Each measurement was performed in triplicate [50].

##### Mechanical Characteristics of In Situ Gelling Formulations

The texture profile analyses of the produced formulations were examined using a CT3 Texture Analyzer (Brookfield, London, UK) to evaluate the mechanical characteristics such as hardness, cohesiveness, adhesiveness, and compressibility. With a set recovery period of 15 s between the end of the first compression and the start of the second, an analytical probe with a diameter of 12.7 cm was immersed into the samples twice to a specified depth of 10 mm, and at a specified rate of 2 mm/s. The applied trigger force was 0.01 N. Triplicate analyses were performed for each sample at 34 ± 1 °C. TexturePro CT V1.6 Build was used for the calculations and data collection. Hardness (the amount of force necessary to cause a particular deformation), compressibility (the work required to deform the product during the probe’s initial pass), adhesiveness (the work required to overcome the attractive forces between the sample’s surface and the probe’s surface), and cohesiveness (the ratio of the area under the curve for the second compression cycle of the force–time curve to the area under the curve for the first compression cycle) could all be determined from the force–time plots that resulted [47].

##### In Situ Gelling Formulation–Mucin Interaction Study

A straightforward approach was used to assess the ‘rheological synergism’ that resulted from mixing mucin dispersions and mucoadhesive in situ gelling formulations containing DGG with the mucoadhesive polymer to measure the mucoadhesive force of the formulations [55]. In particular, it demonstrated more than an additional increase in the viscosity of the mixture that resulted from the interactions between the two macromolecular species’ chains when mucoadhesive polymers and mucin dispersions were mixed. Mucin type II (8%, *w*/*v*) was dissolved in SNF and allowed to equilibrate for at least 2 h. All mucin suspensions were used within 4 h after preparation to prevent mucin degradation. The dispersion was then heated to 34 °C and, after that, was mixed with the formulations (mucin dispersion: in situ gelling formulation ratio: 1:2 (*v*/*v*)) that had also been heated to the same temperature [56,57]. A Brookfield viscometer was used to evaluate the viscosities of the mucin dispersion, formulation, and mucin in situ gelling formulation mixture in triplicate. The mucoadhesion viscosity component was calculated using Equation (1).

η_b_ = η_t_ − (η_m_ + η_p_) (1)

where η_b_ is the viscosity caused by the mucoadhesion, η_t_ is the mixture’s viscosity, η_m_ is the mucin’s viscosity, and η_p_ is the in situ gelling formulation’s viscosity. Equation (2) was used to calculate the mucoadhesive force.

F_b_ = η_b_ × γ (2)

where γ is the shear rate used to determine the viscosity value [56].

##### Ex Vivo Mucoadhesive Strength Test

A tensile test was utilized to assess the ex vivo mucoadhesion of ion-activated in situ gelling formulations using the same probe and texture analyzer as for texture profile analysis. The impact of changing the contact time (1, 2, 3, and 5 min) was examined to optimize the first contact time between the formulations and the nasal mucosa. In order to measure the contact time and mucoadhesive force, formulations were allowed to come into contact with the nasal mucosa. The contact time that yielded the maximum strength was chosen to be the optimum contact time required for sufficient adhesion.

Fresh sheep nasal mucosa was provided from the local slaughterhouse and cleaned with an SNF solution. A thin layer of clean mucosa was placed on the instrument’s test holder [58,59]. Prior to analysis, a thin layer of the respective formulation was formed around the cylindrical probe’s (12.7 mm diameter) surface by immerging it for 10 s in a beaker containing in situ gelling formulations. After being touched on the mucosal surface for 2 min with a compressive energy of 0.5 N, the probe was separated at a speed of 1 mm/s with a triggering force of 3 g. The force required to separate the contact between the mucosa and the probe that contained the formulation was examined using the Texture Pro CT V1.3 Build 15 software [58].

##### Sprayability Analysis

Control of content uniformity and mass uniformity is crucial for ensuring dosage homogeneity in nasal spray treatments. The regulatory framework requires exact controls on dosage content or mass consistency [60]. With this aim, sprayability analysis was performed on the selected in situ gelling formulations according to the literature [61]. According to the Ph. Eur. recommendations, spray bottle pumps were required to be primed five times at intervals of five seconds (or “priming”) before measurements could be taken. After that, a single dose of two puffs was administered, and their unique masses were calculated using different flask weights. This process was also carried out at 7- and 14-day intervals to assess the mass homogeneity of the various in situ gelling formulations at various times of use.

##### Quantitative Determination of 18β-Glycyrrhetinic Acid

A validated RP-HPLC method was used to analyze GA quantitatively, in accordance with a previously reported method [62].

HPLC conditions: The chromatographic analysis was conducted using Shimadzu CTO-20A-type high-performance liquid chromatography (HPLC) with a CTO-20AC column oven, SPD-20A and LC-20AT units, and a Nucleosil^®^ C18 column (5 m, 250 × 4.6 mm). The mobile phase was delivered via isocratic elution at a flow rate of 1.0 mL/min. The mobile phase, which contained methanol and 0.4% phosphoric acid (85:15), was passed through a 0.45 μm membrane before usage. A total of 20 μL was used for the injection volume. The temperature of the column was set at 35 °C, and a wavelength of 251 nm was used.

Preparation of solutions: The solutions were made by solubilizing GA in methanol to obtain 500 ng/mL stock solutions for the HPLC method. The standard working solutions (0.1, 0.5, 1, 2.5, 5, 10, 15, 20, 30 μg/mL) were prepared by diluting the stock solution with methanol.

##### Active Substance Content Determination

The optimized in situ gelling formulation’s drug content was analyzed after mixing the formulation containing 0.5 mg GA with the solvent mixture (methanol and distilled water containing 0.4% phosphoric acid (85:15, *v*/*v*)) in a falcon tube with a volume of up to 25 mL. The mixture was homogenized by vortexing for 5 min. After that, it was sonicated for 1 h and then centrifuged at 4000 rpm for 10 min [63]. The supernatant was filtered (0.45 μm), and the HPLC method is described in the section “Quantitative determination of 18β-glycyrrhetinic acid” was used to determine the concentration of GA.

##### Fourier Transform Infrared Spectrometry (FTIR) Studies

FTIR spectroscopy (Cary 630 FTIR Spectrometer—Agilent, Santa Clara, CA, USA) was used to further assess GA pure substance–excipient interactions. The spectra for GA as a pure substance, and physical mixtures of GA with DGG and Na-CMC (1:1) were analyzed. The IR spectra of the powder samples were scanned from 4000 to 600 cm^−1^.

##### In Vitro Release Study

An in vitro release study was performed using the dialysis bag method, as previously described, for the optimized in situ gelling formulation and the plain in situ gelling formulation (used as a control) [47]. An in situ gelling formulation with a known amount of GA (6 mg) was sealed inside a cellulosic dialysis membrane (Spectra-Por4 dialysis tubing, cut-off 12–14 kDa, Spectrum Laboratories Inc., Piscataway, NJ, USA) and then put into a beaker with 100 mL of SNF solution at pH 5.5 (to mimic the pH of the nasal interstitial fluid). The tests were conducted at a temperature of 34 ± 1 °C, while the system was shaken continually at 100 rpm in a water bath. A total of 1 mL of the release medium was removed and replaced, at specified time intervals, with the same volume of pre-warmed, freshly prepared release solution. The validated HPLC–UV method mentioned in the section “Quantitative determination of 18β-glycyrrhetinic acid” was used to examine the GA content in the samples that were collected.

##### Physicochemical Stability

A sufficient amount of in situ gelling formulation was placed in amber glass bottles and kept for three months under different storage conditions: 5 ± 3 °C, 25 ± 2 °C and 60% RH; and 40 ± 2 °C and 75% RH. Formulations were assessed for their physical appearance, drug content, viscosity, pH, and in situ gelation at predetermined time intervals. All experiments were performed in triplicate [47].

#### 2.2.3. Cell Culture Studies

##### Cells and Virus

Human respiratory syncytial virus (hRSV Long strain: ATCC VR-26) was cultured using human larynx epidermoid carcinoma cells [HEp-2; ATCC (the American Type Culture Collection) CCL 23]. Cells were cultured at 37 °C with 5% CO_2_ in MEM enriched with 10% FBS, 25 μg/mL amphotericin B, 10 mg/mL streptomycin, and 10,000 U/mL penicillin. ATCC-VR-26 coded hRSV was purchased from ATCC and reproduced at Selçuk University’s Science Faculty in the Virology Laboratory. The virus was propagated, as described above, on a 90% confluent cell monolayer in MEM with antibiotics and 2% FBS. The virus titer was calculated using the 50% tissue culture infectious dose (TCID_50_) method and then represented as TCID_50_ per 0.1 mL [64]. The virus was kept at −80 °C prior to use. Ribavirin was used as a positive control for hRSV inhibition. A ribavirin stock solution (1000 g/mL) was prepared using MEM without FBS and kept at −80 °C until use.

##### Cytotoxicity Assay

The XTT-based cell proliferation kit (Catalog no. 20-300-1000), manufactured by the company Biological Industries (Kibbutz Beit Haemek, Israel), was used to examine the cytotoxic effects of ribavirin, GA, the in situ gelling formulation containing GA, and the placebo in situ gelling formulation on HEp-2 cells. According to the manufacturer’s instructions, the tests were conducted as follows: Two-fold decreasing serial dilutions were prepared according to a log2 base from the stock solution of GA using MEM. The final concentrations of the GA dilutions in the wells were 133.3–1.04 µg/mL after 50 µL HEp-2 cell suspensions comprising 2.5 × 10^5^ cells per ml were added. The same processes were applied for ribavirin, the in situ gelling formulation containing GA, and the placebo in situ gelling formulation using another microplate. The final ribavirin concentrations in the wells ranged from 500 to 0.98 µg/mL, while the final GA in situ gelling formulation concentrations in the wells ranged from 33.33 to 0.26 µg/mL. The microplates also included cell control (CC) and media control (MC). The microplates were incubated in a humidified incubator with 5% CO_2_ at 37 °C for 2 days. In each well, 50 µL of a mixture of 0.1 mL PMS activator and 5 mL XTT reagent were then added. After an additional 3 h of incubation, the XTT formazan product was formed in microplates. The average optical densities (OD) from the wells were measured using an ELISA reader (Multiskan EX, Labsystems) at a reference wavelength of 630 nm and a test wavelength of 490 nm. The experiment was carried out in triplicate, and the results were shown as the ratio of the average cytotoxicity to cell control.

The percentage of cytotoxicity of test samples on HEp-2 cells was calculated using the following formula [65]:

$$\mathrm{Cytotoxicity}\;\left(\%\right)=\frac{A-B}{A}\times 100$$

A: The OD of the cell control.

B: The OD for the cells treated with GA, in situ gelling formulations, or ribavirin.

The calculated percentages of cytotoxic effects were graphed against the corresponding concentrations of samples tested (GA, in situ gelling formulations, and ribavirin). The GraphPad Prism 5.03 program was used to analyze the sample concentration that allowed 50% survival of HEp-2 cells (CC_50_) [66]. These calculated CC_50_ values were used to evaluate the antiviral activity of GA, in situ gelling formulations, and ribavirin. The maximum non-toxic concentrations (MNTCs) of GA, in situ gelling formulations, and ribavirin were also calculated by comparing the OD with CC.

##### Antiviral Activity Assay

- Antiviral activity assay in simultaneous treatment with the virus

The anti-RSV activities of GA, in situ gelling formulations, and ribavirin were evaluated using the colorimetric XTT method. The experiment is summarized as follows [67]:

For the experiment, a maintenance medium (MEM with 1% FBS) was used to prepare the RSV suspension at 100 tissue culture infective doses (TCID_50_). The test samples (GA, in situ gelling formulations, and ribavirin) were diluted using a maintenance medium to 2 × MNTCs (MNTCs = 8.34 µg/mL for GA, 4.16 µg/mL for in situ gelling formulations, and 0.98 µg/mL for ribavirin). Following that, from these dilutions, two-fold dilutions were prepared with a maintenance medium. A total of 2.5 × 10^5^ cells were seeded in each well and incubated for 24 h at 37 °C with 5% CO_2_. The production media in the wells was removed once the cells were confluent and, simultaneously, 100 µL of the test samples and 100 µL of the RSV suspension (containing 100 TCID_50_) were both added to the wells. A total of 100 µL of the maintenance medium and 100 µL of the RSV suspension were both added to the virus control (VC) wells. A total of 200 µL of the maintenance medium was placed in the CC wells.

The final concentrations of GA, in situ gelling formulation, and ribavirin were arranged as 8.34–0.07 µg/mL, 4.17–0.03 µg/mL, and 0.980 to 0.004 μg/mL, respectively. Microplates were incubated for 2–5 days at 37 °C with 5% CO_2_, more specifically, until 85–90% of the cytopathic effect (CPE) was developed in the VC wells. When 85–90% of the CPE was seen in the VC wells, the solutions were removed from the wells. Then, in each well, 50 µL of a mixture of 0.1 mL PMS activator and 5 mL XTT reagent were added. The reagent was homogeneously distributed in the wells by gently shaking the microplates. After an additional 3 h of incubation, the XTT formazan product was formed in microplates. The average optical densities (OD) from the wells were measured using an ELISA reader (Multiskan EX, Labsystems) at a reference wavelength of 630 nm and a test wavelength of 490 nm. The protection percentages of GA, in situ gelling formulations, or ribavirin concentrations against viruses were calculated from the following formula [65]:

Protection percentage = [(A − B)/(C − B) × 100]

A = Mean optic density for each GA, in situ gelling formulations, or ribavirin concentration in wells.

B = Virus control OD (average OD values in wells).

C = Cell control OD (average OD values in wells).

Nonlinear regression analysis in GraphPad Prism Version 5.03 was used to calculate the EC_50_ value, which is defined as the concentrations of GA, in situ gelling formulations, or ribavirin that protect 50% of the infected cells, considering the protection rates determined with GA, in situ gelling formulations, or ribavirin concentrations. The selectivity indexes (SI) of GA, in situ gelling formulations, or ribavirin were calculated from the CC_50_/EC_50_ ratio. The experiments were performed in triplicate.

- Pre-infection antiviral activity assay

The test samples (GA and in situ gelling formulations) were diluted using a maintenance medium to 2 × MNTCs (MNTCs = 8.34 µg/mL for GA and 4.16 µg/mL for in situ gelling formulations). Following that, from these dilutions, two-fold dilutions were prepared with a maintenance medium. A maintenance medium (MEM with 1% FBS) was used to prepare the RSV suspension at 100 tissue culture infective doses (TCID_50_). A total of 2.5 × 10^5^ cells were seeded in each well and incubated for 24 h at 37 °C with 5% CO_2_. The production media in the wells was removed once the cells were confluent, and 100 µL of the test samples were added to the wells. A total of 100 µL of the maintenance medium and 100 µL of the RSV suspension were both added to the virus control (VC) wells. A total of 200 µL of the maintenance medium was placed in the CC wells. Then, the microplate was incubated for 1 h at 37 °C with 5% CO_2_. After incubation, 100 µL of the RSV suspension (containing 100 TCID_50_) were added to the wells. The final concentrations of GA were arranged as 8.34–0.07 µg/mL, and the final concentration of in situ gelling formulations was arranged as 4.17–0.03 µg/mL. Microplates were incubated for 2–5 days at 37 °C with 5% CO_2_, more specifically, until 85–90% of the cytopathic effect (CPE) was developed in the VC wells. The XTT measurement procedure was performed, and protection percentages and EC_50_ values were calculated as specified in the Section “Antiviral activity assay in simultaneous treatment with the virus”.

## 3. Results and Discussion

### 3.1. Optimization and Characterization of GA-Loaded In Situ Gelling Formulations

#### 3.1.1. Gelation Capacity of In Situ Gelling Formulations

An in situ gelling system should ideally include a low viscosity fluid to enable reproducible nasal administration, but go through an in situ phase transition to create a gel that can tolerate shear stresses in the nasal passages and maintain drug release under physiological conditions [68]. As a result, extended residence time inside the nasal cavity and less mucociliary clearance would result from the increased viscosity [69]. However, nasal liquid has the potential to dilute DGG and influence the formulation’s ability to gel [63]. To accomplish the necessary ion-sensitive sol–gel transition, the DGG concentration had to be optimized. Therefore, preliminary attempts were undertaken to choose the DGG concentration that provided optimal gelation. Gelation capacity tests were carried out by blending DGG with SNF. The findings showed that a DGG solution with a concentration of more than 0.3% might quickly transform into a colorless and transparent gel. Accordingly, the sol–gel phase change was more favorable with a higher concentration of DGG, suggesting that the gelation characteristics were favorably associated with polymer concentration. However, it was observed that increasing viscosity at higher DGG concentrations would make it difficult to apply as a nasal spray. Therefore, a DGG concentration below 0.7% was considered ideal to avoid discomfort with swelling of the gel after application and to prevent patient non-compliance. The formulation was very easily diluted with SNF, but when the concentration of DGG used was less than 0.4%, it would be challenging to ensure gel formation. This is because nasal fluid dilution has an impact on the gelation capacity. As a result, it was determined that DGG concentration in an in situ gelling formulation should not decrease to 0.4% in our study. Taken together, due to their desirable gelling capabilities and adequate viscosity, DGG concentrations in the range of 0.4–0.6% *w*/*w* were chosen when optimizing the formulation of the intranasal in situ gelling formulation for further studies. These findings were in good agreement with the findings of Hao et al. [64] and Cai et al. [63].

#### 3.1.2. Rheological Evaluation

The most crucial aspects to consider when assessing the effectiveness of in situ gelling systems are gelling capacity and viscosity [8]. The formulation needs to have the ideal viscosity under storage conditions for simple administration into the nasal cavity, and it must quickly convert from sol to gel in contact with SNF to maintain an extended residence time at the administration site.

**Figure 2:**
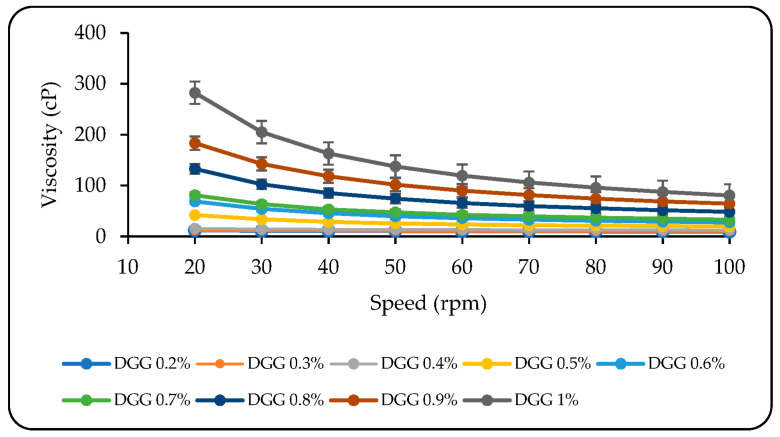
The viscosity values of in situ gelling formulations containing different DGG concentrations at RT (values are expressed as average ± SD) (*n* = 3).

When the sol state rheograms of several DGG formulations were compared, it was determined that concentration-dependent increases in viscosity were seen, which was compatible with the findings of Morsi et al. (Figure 2) [8]. Due to the liquid state of the formulations, mixtures containing 0.2% to 0.6% DGG had low viscosity values at RT. However, the formulations transformed to a low-viscosity gel texture, and their viscosity increased as the DGG concentration increased to 0.7% or higher (Figure 2). In this case, it was considered that the sprayability of the in situ gelling formulations containing DGG at 0.7% and higher concentrations into the nasal passage and the homogeneous spreadability of the formulation on the nasal mucosa may be adversely affected. Therefore, for further research, DGG concentrations in the range of 0.4–0.6% *w*/*w* are preferable due to their appropriate viscosity values.

**Figure 3:**
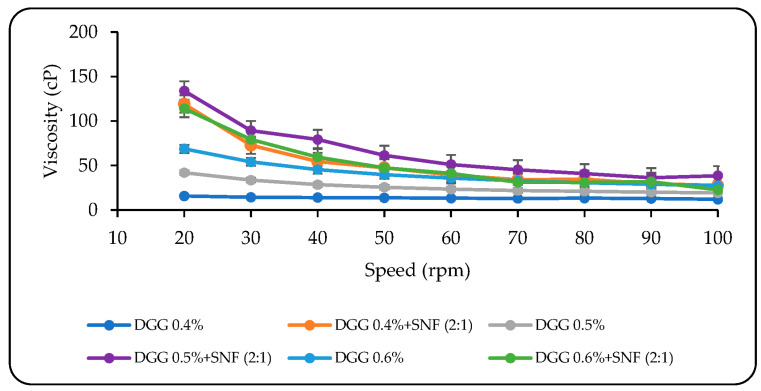
The viscosity values of in situ gelling formulations containing different DGG concentrations before and after mixing SNF solution (values are expressed as average ± SD) (*n* = 3).

On the other hand, adding SNF to all formulations significantly increased the viscosity as a result of gelation (Figure 3). These ion-activated in situ gels behave similar to a non-Newtonian pseudo-plastic fluid with a typical shear thinning feature, since the viscosity of mixtures of the DGG solutions and SNF at 34 °C rapidly reduces depending on the shear rate applied. The potential use of in situ gelling formulations for intranasal administration may greatly benefit from this condition. A pseudo-plastic system generally demonstrates an increase in viscosity after it enters the nasal cavity and comes into contact with the ions present in the nasal fluid, extending the nasal residence time [64]. These results agree with the findings of other studies [60,63,70].

Different polymers are frequently combined to enhance the characteristics of in situ gelling formulations and mucoadhesive compositions. This method can result in improved mucoadhesion or superior gelling characteristics by reducing the amount of polymer in the system [71]. Although DGG has mucoadhesive characteristics, these are inadequate to extend the mucosal residence time as they are dependent on relatively weak forces such as hydrogen bonds and van der Waals forces [72]. Therefore, the optimized selected concentrations of DGG (0.4–0.6%) were combined with four different mucoadhesive polymers (xanthan gum, HPMC, Na-CMC, or Carbopol^®^ 974P NF) to evaluate the potential synergistic impacts brought on by the polymer combinations and to enhance the formulation’s mucoadhesive characteristics.

Mucoadhesion is the term used to describe a material’s ability to adhere to the mucosal membranes of the human body and enable temporary retention. This feature has frequently been used in the development of polymeric dosage forms for drug delivery systems including nasal, buccal, ocular, vaginal, and oral routes. Hydrophilic polymers containing non-ionic functional groups and/or charged groups capable of forming hydrogen bonds with mucosal surfaces typically have excellent mucoadhesive characteristics [73]. Many anionic polymers, including xanthan gum, Na-CMC, and carbomers (weakly cross-linked derivatives of polyacrylic acid) have significant mucoadhesive characteristics due to their capacity to form hydrogen bonds between the glycoproteins in the mucus layer and the carboxylic acid group of polymers [74,75]. Stronger hydrogen bonds cause the delivery system to bond more deeply and strongly to the mucus layer [75]. HPMC is also widely used due to its controlled-release mechanism, in addition to its mucoadhesive capabilities. It has been used to deliver many drugs in various dosage forms. HPMC exhibits less H bonding compared to anionic polymers because of its non-ionic nature and lack of a carboxylic group that gives protons. Most of the time, the specific interactions between mucin and non-ionic polymers are quite weak and frequently cannot be detected by conventional physicochemical methods [76]. Non-ionic polymer-based formulations can exhibit mucoadhesive properties, mainly through the formation of an interpenetrating layer with the mucus gel and the diffusion of its macromolecules [73].

**Figure 4:**
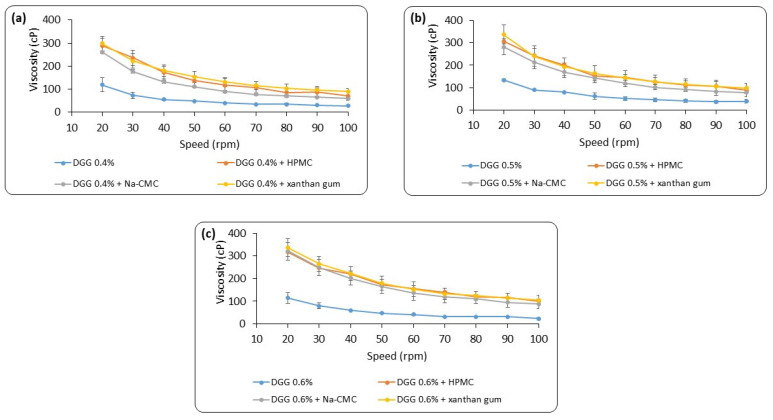
Comparison of the viscosity values of DGG-only and combined in situ gelling mixtures after mixing with SNF (2:1 *v*/*v*); (**a**) 0.4% DGG in situ gelling formulations; (**b**) 0.5% DGG in situ gelling formulations; (**c**) 0.6% DGG in situ gelling formulations (*n* = 3).

As a result of this study, when the viscosity values of in situ gelling formulations containing combined polymers were compared with the viscosity values of in situ gelling formulations containing only DGG, a considerable increase in viscosity was observed. It was found that, according to the type of polymer combined, the increase in viscosity can be ordered as Carbopol^®^ > HPMC > xanthan gum > Na-CMC. The increase in viscosity for the cellulose derivatives could be explained by their ability to form in situ gelling formulations when their aqueous solutions are heated [77]. For Na-CMC, the transition temperature is in the range of 40 to 50 °C, and for HPMC, it is between 75 and 90 °C. The gelation temperature of MC is reported to be lowered to 32–34 °C by adding sodium chloride, while the reduction of the hydroxypropyl molar substitution can lower the HPMC transition temperature to approximately 40 °C [78]. The significantly high viscosity of xanthan gum, on the other hand, can be linked to its anionic character and is known as an ion-driven in situ gelling polymer. Its molecules become extended due to the electrostatic repulsions caused by the charged groups on the side chains [79]. In turn, this causes the molecules to align and join together through hydrogen bonds to create a weakly organized helical conformation that would immobilize free water and enhance viscosity [79]. A well-known polymer that induces in situ gelling due to pH is Carbopol^®^. It is a polyacrylic acid polymer that, when the pH is raised above its pK of around 5.5, exhibits a sol–gel phase transition in an aqueous solution [80]. At low pH values and high pH values, the carboxylic groups of polyacrylic acid (PAA) receive and release protons, respectively. The electrostatic repulsion of the negatively charged groups causes the PAA to swell and expand up to 1000 times its initial volume at a high pH [81]. In our study, the combination of Carbopol^®^ with different DGG concentrations demonstrated the highest increase in viscosity when mixed with SNF at a 0.5% Carbopol^®^ concentration. However, in situ gelling formulations containing Carbopol^®^ are too viscous, and their consistency is not uniform. Therefore, they were excluded from further studies as they are thought to cause discomfort in the nasal cavity and cannot ensure dose homogeneity at the application site. Data on the comparison of viscosity values of in situ gelling formulations containing combined polymers, except for Carbopol^®^, with those containing only DGG are given in Figure 4.

#### 3.1.3. Mechanical Characteristics of In Situ Gelling Formulations

The development of topical intranasal formulations faces considerable difficulty in achieving a number of anticipatory properties, including improved spreadability, good mucoadhesion, and appropriate viscosity, to support comfortable administration and patient compliance [82]. By examining the physical gel structure, texture profile analysis (TPA) enables the evaluation of the mechanical characteristics of semi-solid formulations. The mechanical characteristics (hardness, compressibility, adhesiveness, and cohesiveness) of in situ gelling formulations using mixed polymers were determined from the force–time curve that resulted from TPA diagrams.

One of the important mechanical parameters is adhesiveness because it provides optimal gel contact and retention at the mucosal surface, resulting in increased medication bioavailability [47]. When compared to neutral polymers, anionic polyelectrolytes have been determined to generate stronger adhesions with the mucus layer’s glycoprotein chains because charged functional groups present in the polymer chain significantly affect the bioadhesion strength [83]. Similar results were obtained in this study. The mucoadhesive polymers can be aligned based on their adhesiveness at a 0.5% concentration of DGG and mucoadhesive polymer as follows: Na-CMC (0.300 ± 0.05 mJ) > xanthan gum (0.100 ± 0.00 mJ) > HPMC (0.050 ± 0.05 mJ). This may clarify the comparatively weak mucoadhesive strength of non-ionic HPMC in comparison with anionic Na-CMC and xanthan gum [50]. Furthermore, the hardness and compressibility values were quite similar between different mucoadhesive polymers, and it was observed that the hardness of the in situ gelling formulations was directly related to their viscosity, since an increase in gel viscosity was correlated with an increase in hardness. This is consistent with the in situ gelling formulation’s extent of crosslinking. A greater crosslink quantity per unit volume results in greater gel strength values and, consequently, higher viscosities in the resulting gels [84]. On the other hand, lower values for cohesiveness denote better spreadability [54]. In this study, cohesiveness at a 0.5% concentration of DGG and mucoadhesive polymers was as follows: HPMC (0.915 ± 0.05) > xanthan gum (0.410 ± 0.06) > Na-CMC (0.140 ± 0.05).

#### 3.1.4. In Situ Gelling Formulation–Mucin Interaction Study

The current experiment relied on the hypothesis that rheological synergism results from chemical interactions and entanglements between components of the formulation and glycoproteins in mucus [55]. Commercial mucin, the primary component of mucus, was used in this test to assess the formulations’ mucoadhesion. When mucoadhesive polymers and mucin dispersions are combined, a rheological synergism may be observed based on the interactions between the chains of the two macromolecular species. This indicates a more than additive growth of the mixture’s viscosity. In other words, the equation [η_mixture_ − (η_polymer_ + η_mucin_)] > 0 denotes the additional viscosity-enhancing effect of the mucin–polymer interaction relative to the value predicted based on the contributions of the mucin and polymer, which are simply additive [85].

As can be seen in Table 4, at the examined shear rate (34 s^−1^), the viscosity values (cP) of the mixes were greater than the total values of mucin and the corresponding in situ gelling formulation. This would suggest rheological synergism between the in situ gelling formulation and mucin dispersions rather than additive growth. One explanation might be the development of secondary chemical interactions and molecular entanglements between gel solutions and the mucus glycoproteins [55]. The formulation containing Na-CMC demonstrated substantial synergism, and it was determined that, as the DGG concentration increased, the mucoadhesive force (Fb) values increased proportionally. This was possibly anticipated because the greatest mucoadhesive qualities are found in the large families of hydrophilic polymers that include the carboxylic group [86].

Considering all of the data obtained thus far, the in situ gelling formulation containing 0.5% DGG + Na-CMC polymers was in clear liquid form at RT and was considered to have a sprayable viscosity compared to the 0.6% DGG + Na-CMC formulation. When mixed with SNF at 34°C, the 0.5% DGG + Na-CMC formulation’s viscosity increased significantly (before gelation: 74.23 cP; after gelation: 269.55 cP) and formed a clear gel. As a result of TPA analysis, the 0.5% DGG + Na-CMC formulation was found to have a higher adhesiveness value (0.300 mJ) compared to other in situ gelling formulations containing HPMC and xanthan gum polymers. Furthermore, it had the lowest cohesiveness value (0.140 ± 0.05), providing better spreadability of the formulation on the nasal mucosa. Moreover, the mucoadhesive force (9.39 Pa) was determined to be superior to that of the other in situ gelling formulations in the rheological synergism study. Considering all of the data, it was decided to continue further studies with the formulation containing 0.5% DGG + Na-CMC in combination.

In the subsequent study, an investigation was carried out to determine whether different concentrations of Na-CMC, as the selected mucoadhesive polymer, affected the mucoadhesive force of the in situ gelling formulations. For this purpose, in situ gelling formulations containing varied concentrations of Na-CMC (0.1, 0.3, 0.5 and 0.7% *w*/*v*) and DGG (0.5% *w*/*v*) were prepared, and the in situ gelling formulation–mucin interaction was investigated. Based on the results, it was observed that all of the formulations containing Na-CMC showed positive synergism values; this indicated that the observed viscosity for the mucin-containing blends was greater than the total of the measured viscosities for the in situ gelling formulation and the mucin solution. The degree of synergistic increase was related to the concentration of Na-CMC (Table 5). The synergism of the formulation increased along with the increase in Na-CMC concentration.

#### 3.1.5. Ex Vivo Mucoadhesive Strength Test

Utilizing polymers with substantial mucoadhesive properties can dramatically reduce the formulation’s total clearance from the nasal cavity, resulting in a longer retention time and improved bioavailability of the medication [47]. To compare the ex vivo mucoadhesive strength of in situ gelling formulations containing various Na-CMC concentrations, the ex vivo mucoadhesive strength test was carried out.

The in situ gelling formulations had adhesive qualities that increased with the concentration of gelling agent (Na-CMC) from 0.1 to 0.7%, according to an assessment of the mucoadhesive strength in terms of detachment stress (Table 6). These results agreed with the findings of other studies [47,58].

#### 3.1.6. Sprayability

The findings of the spray uniformity tests are displayed in Table 7. The weight deviations (%) of the mean values of T7 (the 7th day of the experiment) and T14 (the 14th day of the experiment) from the target values (mean values of T0 (the first day of the experiment)) remained below 2% for all in situ gelling formulations. These deviations were found to be in accordance with the Ph. Eur. and the FDA draft guidelines. The Ph. Eur. criteria state that no more than two values may vary from the mean value by more than 25% and none may vary by more than 35%. According to recently proposed guidelines from the FDA, the weight of each spray should not exceed 15% of the target weight, and the mean weight should not exceed 10% of the target weight [61]. Considering usage at 7th- and 14th-day intervals, all formulations met these specifications. Although the formulation containing 0.7% Na-CMC was found to comply with the sprayability standards, the spreadability of the in situ gelling formulation droplets during spraying was less compared to other in situ gelling formulations. Therefore, the formulation prepared with 0.7% Na-CMC was not found to be suitable for administration as a nasal spray and was eliminated from the study.

#### 3.1.7. Quantitative Determination of 18β-Glycyrrhetinic Acid

**Figure 5:**
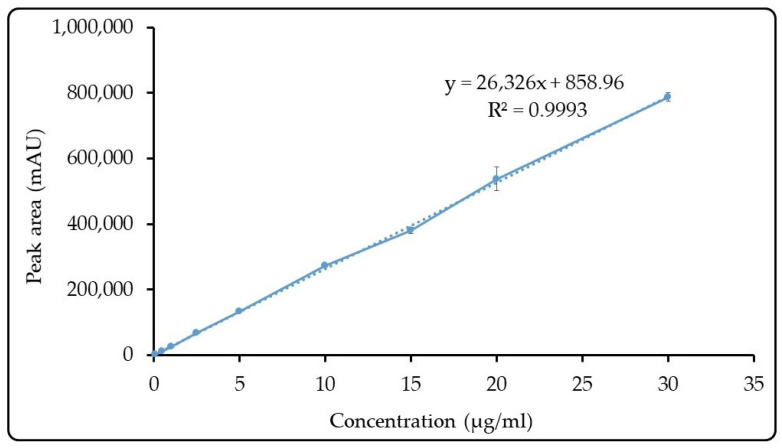
Calibration curve of GA (means ± SD, *n* = 6).

The quantitative analysis of GA was accomplished by the validated HPLC–UV method in accordance with ICH recommendations for the active substance content determination and in vitro release study. Without interference from impurities in the analyzed matrices, GA was eluted in 10.7 min. The response of the detector was linear throughout a range of 0.10–30 g/mL. The method showed excellent accuracy (recovery% ranged from 101.16 to 103.67%), and intraday and interday precision (RSD% values were 0.44% and 0.35%, respectively) (*n* = 6). The regression equation was y = 26326x + 858.96 and the coefficient of determination was R^2^ = 0.9993. Figure 5 shows a plot of the findings from the linearity study.

#### 3.1.8. Active Substance Content Determination

Each unit in a batch needs to have a drug substance quantity that is tightly confined to the label claim to ensure that dosage units are uniform. One dose or a proportion of a dose of a drug substance is found in each dosage unit, which is also known as a dosage form. The definition of “dosage unit uniformity” is the level of uniformity at which the drug material is distributed throughout the dosage units. Testing for uniformity of dosage units is specified by the USP 44 − NF 39 <601> Inhalation and Nasal Drug Products: Aerosols, Sprays, and Powders—Performance Quality Tests. If the amount of the drug substance is between 85.0% and 115.0% of the label claim, no unit is outside of the range of 75.0% to 125.0% of the label claim, and the RSD of the 10 dosage units is less than or equal to 6.0%, the dosage uniformity standards are met [87]. In our study, the percentage active substance content and the RSD of the samples for the optimized formulation were determined to be satisfactory in the range of 100.46–101.78% and equal to 0.49%, respectively.

#### 3.1.9. Fourier Transform Infrared Spectrometry (FTIR) Studies

**Figure 6:**
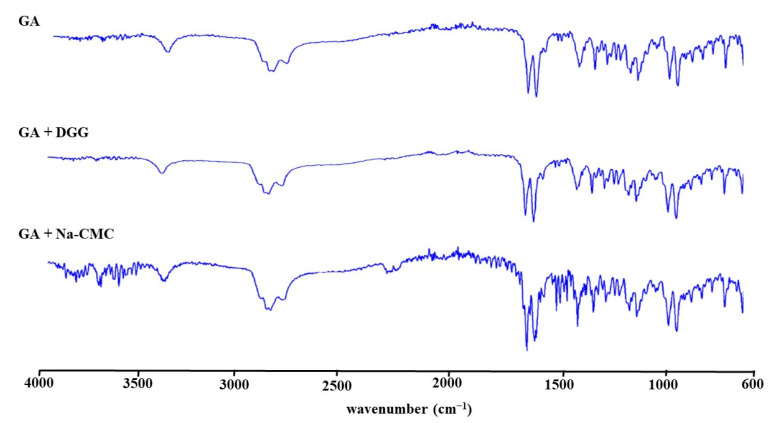
FTIR spectra of pure GA, physical mixtures of DGG, and Na-CMC with pure GA.

FTIR analysis was carried out to determine whether the active ingredient molecule had undergone any chemical alteration as a result of the excipients. The distinctive absorption bands linked to the stretching vibration of the carbonyl group in carboxyl groups and ketone groups showed at 1704 and 1664 cm^−1^, respectively, in the FTIR spectra of GA (Figure 6). The results revealed that the FTIR spectra of GA in the physical mixtures were identical to those seen for pure GA, proving that the excipients utilized had no impact on the chemical stability of GA.

#### 3.1.10. In Vitro Release Study

A cornerstone of treatment for the prevention of virus transmission is a nasal spray [88]. However, the physiological mechanism most closely associated with the lowering of the active substance residence time in the nasal cavity is mucociliary clearance. This self-clearing process is responsible for the rapid elimination of the active substance from the nasal cavity, which shortens the time it takes for the active substance to exert its therapeutic effects [89]. To prevent the rapid drainage of drugs and extend their residence time in the nasal cavity when applied as conventional aqueous solutions, a viscosity-increasing strategy has been suggested: nasal in situ gelling formulations appear to be a better option than nasal liquid ones [90]. These formulations are simple to administer as low-viscosity polymeric solutions, allowing ideal nasal accumulation. When these solutions come into contact with the mucosa, they transform into gels. The development of a polymeric network in vivo ensures the continuous release of a medicinal substance and extends the amount of time that the drug is in contact with the site of action or absorption [39].

**Figure 7:**
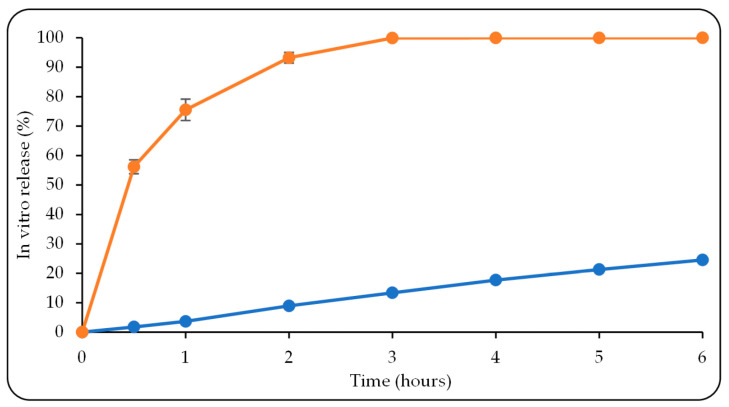
In vitro GA release study of the optimized in situ gelling formulation (blue line) and a GA suspension as a control (orange line). Data were reported as mean ± SD, (*n* = 3).

To identify the GA release pattern from the optimized in situ gelling formulation and to predict the formulation’s ability for controlled release after application to the nasal cavity, an in vitro drug release study was carried out compared to that of a GA suspension as a control. As is seen in Figure 7, after 6 h, 99.85 ± 0.68% of the GA was determined to have been released within 3 h, since the control sample was made by dissolving GA as a saturated solution in distilled water. Conversely, GA release from the in situ gelling formulation reached only 24.56 ± 0.49%, indicating the prolonged GA release from the optimized formulation over time. The viscous nature of the in situ gelling formulation could be a possible explanation for this sustained-release behavior [91].

#### 3.1.11. Physicochemical Stability

Before using formulations in the relevant field, their stability needs to be assessed. However, for the proper and timely completion of the product development phase, a rapid assessment of stability is essential. For a formulation to maintain its integrity throughout the course of its shelf life and to be resistant to environmental factors such as heat and humidity, it must be produced using suitable manufacturing processes and have a well-designed composition [68]. In this context, to examine the physicochemical stability of the optimized in situ gelling formulation, samples underwent examinations for their appearance, active substance content, pH, viscosity, and in situ gelation at designated time points (on the day the formulation was prepared, and the first, second, and third months after production) under three different stability test conditions (at 4 °C; 25°C and 60% relative humidity; and 40 °C and 75% relative humidity). As a result, it was determined that the optimized formulation’s physical appearance was unaffected by time or temperature since it remained a clear liquid at different storage conditions for three months.

The systemic approach to stability evaluation includes information on the stability of the active substance as a key component. The minimum permissible potency level is typically accepted to be 90% of the labeled potency, according to the Food and Drug Administration (FDA) Drug Stability Guidelines [69]. With respect to drug content, the obtained results (96.44 ± 0.06% for 4 °C, 98.37 ± 0.13 for 25 °C, and 94.14 ± 0.15 for 40 °C) at the end of the third month were almost constant compared to the initial drug content (Table S1). A neutral to slightly acidic pH is well tolerated because the nasal mucosa has a pH of approximately 5–6.5 [70]. With respect to pH, the in situ gelling formulation did not exhibit any notable alterations during the study’s duration (initial pH value: 6.47 ± 0.01; pH values after the third month: 6.46 ± 0.01, 6.47 ± 0.01, and 6.45 ± 0.01 at 4, 25, and 40 °C, respectively) (Table S1).

An in situ nasal gel should have the ideal viscosity for simple nasal usage as a liquid that transforms into gel form under intrinsic conditions in the nose. Additionally, the gel should maintain its form to allow for longer contact between the medication and the nasal cavity’s absorptive sites and to limit formulation drainage to enable continuous delivery of the active substance [47]. From viscosity and in situ gelation analyses, it was demonstrated that all samples were in liquid form under storage conditions and were turned into a gel after mixing with SNF at 34 °C, consistent with the initial viscosity values (Table S2).

### 3.2. Cell Culture Studies

#### 3.2.1. Cytotoxicity Assay

The cytotoxic effects of GA, the in situ gelling formulation containing 0.1% (*w*/*w*) GA and a 0.5% (*w*/*w*) DGG + 0.5% (*w*/*w*) Na-CMC combination, the placebo in situ gelling formulation (same formulation without GA), and ribavirin on HEp-2 cells were assessed by a colorimetric cell viability test. In the experiments, the non-toxic dose of the test samples and ribavirin on HEp-2 cells was found. The obtained MNTCs and CC_50_ values of the test samples and ribavirin against HEp-2 cells are demonstrated in Table 8.

The CC_50_ and MNTC values of GA, the in situ gelling formulation containing GA, the placebo in situ gelling formulation, and ribavirin were determined as 47.59, 15.29, 14.84, and 117.00 µg/mL and 8.33, 4.16, 4.16, and 0.98 µg/mL, respectively.

#### 3.2.2. Virus Titration

**Figure 8:**
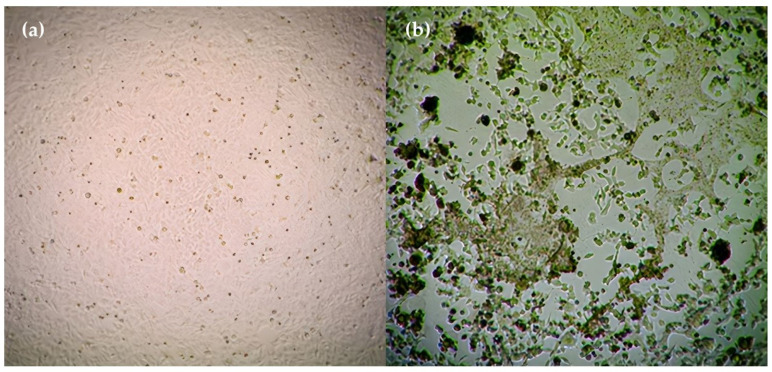
(**a**) Image of HEp-2 cells; (**b**) CPE image of RSV in HEp-2 cells (original), (4× magnification).

In the titration of RSV in the HEp-2 cell culture using the microtitration method, virus titers were found to be TCID_50_ = 10–4.5/0.1 mL at the end of the third day. The CPE of the virus in HEp-2 cells, and the appearance of uninfected HEp-2 cells (HEp-2 control) are displayed in Figure 8.

#### 3.2.3. Antiviral Activity Assay

The EC_50_ and SI values of the test samples and ribavirin obtained as a result of the antiviral assays are shown in Table 9.

According to the antiviral activity study, it was found that GA and the in situ gelling formulation containing 0.1% (*w*/*w*) GA and a 0.5% (*w*/*w*) DGG + 0.5% (*w*/*w*) Na-CMC combination had substantial antiviral activity that was superior to ribavirin (used as a positive control). Furthermore, it can be seen that (Table 7) GA and the in situ gelling formulations were less toxic compared to ribavirin on HEp-2 cells, and the CC_50_ values of GA, in situ gelling formulations, and ribavirin were higher than the EC_50_ values.

Chattopadhyay et al. (2009) stated that if the SI value obtained according to the antiviral result is 10 or greater than 10, substances may have potential antiviral activity [71]. Considering this, it was observed that the antiviral activity of the in situ gelling formulation developed using 18 β-GA was high in the protection of cells before active infection of hRSV, especially in terms of the prevention of hRSV entry into the cell. The binding glycoprotein (G) and fusion (F) glycoprotein on the surface of the virion control the first stage of hRSV infections [72]. G glycoprotein interacts with host cell receptors and allows adsorption to the cell surface [73]. G glycoprotein targets receptors in ciliated cells of the air passages, while F glycoprotein leads to the virion membrane fusing with a target cell membrane [72,73]. Therefore, it was thought that both 18 β-GA and the in situ gelling formulation containing 18 β-GA could significantly inhibit the binding and adsorption of hRSV to the host cell. However, the inverse relationship between GA and the SI values for the GA in situ gelling formulation for the simultaneous (SI value of GA < SI value of in situ gelling formulation) and pre-infection (SI value of GA > SI value of in situ gelling formulation) applications could be attributed to the fact that DGG in the in situ gelling formulation was applied before the infection initially interacted with the cations in the cell culture medium and transformed to a gel but lost its gel structure over time (after 1 h) due to the dilution with the cell culture medium. Therefore, it was considered that it was not able to block the binding of the virus to the cell receptors as effectively as the simultaneous application. However, since high SI values were obtained with both pre-infection and simultaneous applications of the in situ gelling formulation, it was concluded that the optimized formulation showed antiviral activity in both applications.

## 4. Conclusions

In our study, for the first time, a stable in situ gelling formulation containing a polymer combination of 0.5% *w*/*w* DGG and 0.5% *w*/*w* Na-CMC and 0.1% *w*/*w* GA (a promising antiviral substance against RSV) with a good rheological synergism (mucoadhesive force value of 9.39 Pa), a high adhesiveness value of 0.300 mJ, an ex vivo mucoadhesive strength of 19.67 g, a drug content uniformity of 0.494 (RSD%), and sustained drug release over a period of 6 h (24.56 ± 0.49%) was developed for nasal administration against RSV infection. Additionally, antiviral activity was monitored indirectly by the colorimetric XTT method. According to the results, the antiviral activity of the optimized in situ gelling formulation containing GA was shown to be highly effective in inhibiting infection in the HEp-2 cell line (SI value of the in situ gelling formulation = 306 for simultaneous treatment with the virus; SI value of the in situ gelling formulation = 100 for pre-infection application). Therefore, it is considered that the developed in situ gelling formulation is a promising non-invasive, cost-effective, easy-to-use, and self-administrable drug delivery system with high potential to be applied as an alternative antiviral formulation against RSV infection.

## Figures and Tables

**Table 1:** Composition of in situ gelling formulations containing various concentrations of DGG.

Ingredients	Composition (% *w*/*w*)								
DGG	0.2	0.3	0.4	0.5	0.6	0.7	0.8	0.9	1
GA	0.1	0.1	0.1	0.1	0.1	0.1	0.1	0.1	0.1
Dexpanthenol	0.2	0.2	0.2	0.2	0.2	0.2	0.2	0.2	0.2
Glycerin	1	1	1	1	1	1	1	1	1
Benzalkonium chloride	0.02	0.02	0.02	0.02	0.02	0.02	0.02	0.02	0.02
Distilled water	98.48	98.38	98.28	98.18	98.08	97.98	97.88	97.78	97.68

**Table 2:** Composition of different in situ gelling formulations using combined polymers.

Ingredients	Composition (% *w*/*w*)											
DGG	0.4	0.5	0.6	0.4	0.5	0.6	0.4	0.5	0.6	0.4	0.5	0.6
GA	0.1	0.1	0.1	0.1	0.1	0.1	0.1	0.1	0.1	0.1	0.1	0.1
Xanthan gum	0.5	0.5	0.5	-	-	-	-	-	-	-	-	-
HPMC	-	-	-	0.5	0.5	0.5	-	-	-	-	-	-
Na-CMC	-	-	-	-	-	-	0.5	0.5	0.5	-	-	-
Carbopol^®^ 974P NF	-	-	-	-	-	-	-	-	-	0.5	0.5	0.5
Dexpanthenol	0.2	0.2	0.2	0.2	0.2	0.2	0.2	0.2	0.2	0.2	0.2	0.2
Glycerin	1	1	1	1	1	1	1	1	1	1	1	1
Benzalkonium chloride	0.02	0.02	0.02	0.02	0.02	0.02	0.02	0.02	0.02	0.02	0.02	0.02
Distilled water	97.78	97.68	97.58	97.78	97.68	97.58	97.78	97.68	97.58	97.78	97.68	97.58

**Table 3:** Composition of different in situ gelling formulations using Na-CMC polymer.

Ingredients	Composition (% *w*/*w*)			
DGG	0.5	0.5	0.5	0.5
GA	0.1	0.1	0.1	0.1
Na-CMC	0.1	0.3	0.5	0.7
Dexpanthenol	0.2	0.2	0.2	0.2
Glycerin	1	1	1	1
Benzalkonium chloride	0.02	0.02	0.02	0.02
Distilled water	98.08	97.88	97.68	97.48

**Table 4:** Viscosity (η_p_, η_m_, η_t_, η_b_) and mucoadhesive force (Fb) values of the combined in situ gelling formulations were calculated at a shear rate value equal to 34 s^−1^ (*n* = 3).

Formulation	η_p_	η_m_	η_t_	η_b_	Fb (Pa)
DGG 0.4% − HPMC	125.68 ± 2.84	13.95 ± 0.32	238.13 ± 11.25	98.50 ± 2.76	3.94 ± 0.45
DGG 0.5% − HPMC	168.68 ± 6.23	13.95 ± 0.32	294.35 ± 14.98	111.73 ± 4.87	4.47 ± 0.87
DGG 0.6% − HPMC	251.36 ± 16.89	13.95 ± 0.32	343.96 ± 10.62	78.65 ± 1.34	3.15 ± 0.23
DGG 0.4% − Na-CMC	55.59 ± 2.38	13.95 ± 0.32	191.82 ± 7.94	122.28 ± 8.98	4.89 ± 0.98
DGG 0.5% − Na-CMC	72.48 ± 1.68	13.95 ± 0.32	321.08 ± 9.15	234.65 ± 11.85	9.39 ± 0.34
DGG 0.6% − Na-CMC	74.01 ± 2.24	13.95 ± 0.32	327.43 ± 12.18	239.46 ± 10.37	9.58 ± 1.36
DGG 0.4% − xanthan gum	99.22 ± 3.34	13.95 ± 0.32	317.51 ± 15.64	204.34 ± 8.82	8.17 ± 1.67
DGG 0.5% − xanthan gum	148.83 ± 5.75	13.95 ± 0.32	317.04 ± 10.21	154.26 ± 2.75	6.17 ± 1.43
DGG 0.6% − xanthan gum	188.52 ± 4.90	13.95 ± 0.32	317.51 ± 6.86	115.04 ± 9.46	4.60 ± 0.24

**Table 5:** Viscosity (η_p_, η_m_, η_t_, η_b_) and mucoadhesive force (Fb) values of the in situ gelling formulations containing different concentrations of Na-CMC, calculated at a shear rate value equal to 34 s^−1^ (*n* = 3).

Formulation	η_p_	η_m_	η_t_	η_b_	Fb (Pa)
DGG 0.5% − Na-CMC 0.1%	20.28 ± 0.33	16.51 ± 0.49	298.84 ± 18.36	262.06 ± 13.36	8.91 ± 1.21
DGG 0.5% − Na-CMC 0.3%	54.28 ± 0.71	16.51 ± 0.49	315.03 ± 18.39	244.23 ± 11.36	8.30 ± 0.56
DGG 0.5% − Na-CMC 0.5%	74.23 ± 3.16	16.51 ± 0.49	371.08 ± 19.17	280.34 ± 16.23	9.53 ± 0.78
DGG 0.5% − Na-CMC 0.7%	116.41 ± 5.82	16.51 ± 0.49	371.37 ± 15.89	238.44 ± 10.20	8.11 ± 0.43

**Table 6:** Mucoadhesive strength values of in situ gelling formulations containing different Na-CMC concentrations (*n* = 3).

Formulation	Adhesion Strength (g)
DGG 0.5% − Na-CMC 0.1%	14.33 ± 8.40
DGG 0.5% − Na-CMC 0.3%	15.67 ± 5.20
DGG 0.5% − Na-CMC 0.5%	19.67 ± 5.90
DGG 0.5% − Na-CMC 0.7%	27.67 ± 6.80

**Table 7:** Average variation in weight (weight in mg) and weight deviations (%) with a weekly application (*n* = 3).

Formulation	T1	T7		T14	
	Mean ± SD	Mean ± SD	Weight Deviation (%)	Mean ± SD	Weight Deviation (%)
DGG 0.5% − Na-CMC 0.1%	0.323 ± 0.003	0.319 ± 0.002	1.24	0.320 ± 0.003	0.93
DGG 0.5% − Na-CMC 0.3%	0.319 ± 0.002	0.326 ± 0.001	2.19	0.323 ± 0.002	1.25
DGG 0.5% − Na-CMC 0.5%	0.306 ± 0.001	0.299 ± 0.002	2.29	0.302 ± 0.001	1.30
DGG 0.5% − Na-CMC 0.7%	0.299 ± 0.003	0.287 ± 0.001	4.01	0.294 ± 0.001	1.67

**Table 8:** The cytotoxicity results for GA, the in situ gelling formulation, and ribavirin (*n* = 3).

	Cytotoxicity	
Sample Type	MNTC ^a^ (µg/mL)	CC_50_^b^ (µg/mL)
GA	8.33	47.59
GA in situ gelling formulation	4.16	15.29
Placebo in situ gelling formulation	4.16	14.84
Ribavirin	0.98	117.00

^a^ MNTC: The maximum non-toxic concentration; ^b^ CC_50_: 50% cytotoxic concentration.

**Table 9:** The antiviral activity results for GA, in situ gelling formulations, and ribavirin (*n* = 3).

Sample Type	Simultaneous		Pre-Infection	
	EC_50_^a^ (µg/mL)	SI ^b^	EC_50_^a^ (µg/mL)	SI ^b^
GA	0.435	109.65	0.115	415.00
GA in situ gelling formulation	0.050	306.00	0.154	100.00
Placebo in situ gelling formulation	0.790	18.83	2.005	7.40
Ribavirin	4.189	28.00		

^a^ EC50: Half maximal effective concentration; ^b^ SI: Selectivity index.

## Data Availability

Not applicable.

## Supplementary Materials

The following supporting information can be downloaded at: https://www.mdpi.com/article/10.3390/pharmaceutics15082055/s1, Table S1: Stability study results of pH and drug content% at 4, 25, and 40 °C; Table S2: Stability study results of viscosity measurements at 4, 25, and 40 °C.
